# Evaluation of the clinical use of a digital support app for employees with musculoskeletal pain and their employers in an industrial workplace. A mixed methods study

**DOI:** 10.1177/20552076251342014

**Published:** 2025-05-14

**Authors:** Gunilla M Liedberg, Mathilda Björk, Linn Karlsson, Christina Turesson

**Affiliations:** 1 Department of Health, Medicine and Caring Sciences, Division of Prevention, Rehabilitation and Community Medicine, 4566 Linköping University, Linköping, Sweden; 2 Pain and Rehabilitation Centre, and Department of Health, Medicine and Caring Sciences, 4566 Linköping University, Linköping, Sweden

**Keywords:** workplace engagement, worker participation, mobile phone use, prevention, communication, collaboration

## Abstract

**Background:**

Chronic pain affects 20% of Europe's population, reducing work capacity and quality of life. Despite its impact, few digital tools support pain management or employer-employee collaboration. Sustainable WorkEr, a digital support for Persons with chronic Pain and their Employers (SWEPPE), a digital intervention, helps employers sustain the employment of individuals with musculoskeletal pain through workplace support.

**Aim:**

This mixed methods evaluation aims to assess the experiences of employees dealing with musculoskeletal pain, as well as their employers, in using SWEPPE on-site at the workplace for seven months, and to explore how they specifically utilised the app.

**Methods:**

Ten employers and 15 employees at a Swedish industrial company were recruited for this study. Employees used the SWEPPE app alongside regular rehabilitation, while employers accessed it via a web platform. Data were gathered through an HR-developed questionnaire, interviews by two researchers, and user data from the SWEPPE database.

**Results:**

SWEPPE improved communication and collaboration between employees and employers through structured updates and information-sharing. Employers appreciated the insights, enhancing their understanding of employees’ challenges and making meetings more productive. Access to employees’ goals and strategies streamlined processes, increasing awareness of how pain affected work ability and fostering a more supportive work environment.

**Conclusions:**

The SWEPPE app helped employees track their health, work ability, and goals, increasing their awareness of how symptoms affected their work. It fostered structured employee-employer dialogue, enabling collaborative plans that supported both employee recovery and employer readiness for the RTW process. This proactive approach encouraged sustainable workplace adjustments, promoting work sustainability.

## Introduction

Chronic musculoskeletal pain (CMSP), defined as pain persisting for over three months, encompasses conditions such as chronic neck and shoulder pain, back pain, and generalized widespread pain, including fibromyalgia (FM).^1,2^ Initially, pain may be localized but can progressively spread, emphasizing the importance of early intervention to prevent long-term suffering and mitigate its impact on work capacity.^3^ This condition often leads to sick leave, reduced productivity, and additional costs such as overtime, replacement wages, equipment modifications, and training for new employees.^4^ Moreover, CMSP significantly diminishes patients’ quality of life and functional abilities.^5^

Studies have shown prevalence rates of CMSP between 15.9%^6^ and 18.6%^7^ with the highest occurrence among individuals aged 30 to 50 years.^6^ Given the strong correlation between musculoskeletal pain and work behavior, implementing preventive measures is crucial to protect employees from developing these conditions.^7^

Many workers experience manageable CMSP that, if addressed, can prevent long-term sick leave. While some are already on sick leave, others continue to work despite their pain, staying at work (SAW), demonstrating high levels of sickness presenteeism – working while unwell.^8^ Those who engage in presenteeism or take extended sick leave are at risk of developing persistent and widespread pain.^9^ Presenteeism leads to diminished productivity,^10^ as employees struggle with task execution, maintaining work pace, and energy levels due to pain.^11–13^ This behaviour is costly for employers and increases the risk of future health issues and sick leave.^11–13^

The duration of sick leave is a key predictor of future work absences, making it crucial to reduce these periods for the benefit of both individuals and society.^14^ Compared to absentees, presentees are more likely to respond positively to workplace interventions and return to full-time work^11^ or a work situation that is sustainable for the individual. This suggests that adapting to the work environment and proactively addressing health issues can benefit both the employee and the employer. However, a lack of employer support,^15^ often stemming from insufficient knowledge, hinders effective adaptation of work for affected employees.^16^

The European Agency for Safety and Health at Work's^17^ current initiative underscores the need for innovative strategies to support the growing number of Europeans with musculoskeletal disorders, including pain, in remaining productive. Key components include early employer-initiated contact, seamless coordination of the return-to-work (RTW) process, employer education, and collaboration among employers, healthcare providers, and workers. In 2019, the Swedish Association of Local Authorities and Regions (SALAR) increased the responsibility of employers to facilitate continued employment of individuals experiencing illness, emphasising the significance of employers possessing adequate knowledge of pain conditions and implementing effective workplace strategies.^18^ Support and collaboration are recognised as crucial for sustaining long-term employment and creating a stable work environment.^19–21^

Simple self-reporting methods in the workplace are valuable for identifying key individual, psychosocial and work-related factors to prevent pain from becoming chronic.^22^ A digital application for employees with pain conditions can facilitate early intervention by clarifying how symptoms affect work, identifying issues, and providing improvement strategies. A digital support enhances knowledge, access to information, and communication.^23^ Key factors for successful digital support in managing CMSP include empowering individuals through self-management and motivation.^23^ Essential features include access to downloadable online material,^24^ communication with a coach,^25,26^ and setting personal goals, including timelines and plans for RTW.^27^ Self-assessment of symptoms for ongoing health monitoring is also crucial.^27^

To address these key factors, the digital tool SWEPPE was developed. This innovative intervention was designed to assist individuals with chronic pain in transitioning back to work and creating a sustainable work environment. SWEPPE was developed through an agile, user-centred process involving both individuals with chronic pain and experienced employers.^28^ Previous user tests have shown that SWEPPE is user-friendly, relevant and offers valuable support to both employers and workers.^29^ A feasibility study^30^ highlights SWEPPE's ease of use, its role in enhancing knowledge and understanding, and its ability to improve goalsetting, strategies, and collaboration between employers and employees. Additionally, an observational study^31^ revealed that participants utilized various functions, including daily self-registration, goal setting, self-monitoring and identifying employer support. This demonstrates the flexibility of SWEPPE, allowing individuals to choose functions that best meet their needs. Further, an ongoing RCT is evaluating SWEPPE's effectiveness in reducing sick leave during the first-year post-rehabilitation for patients who have completed an interdisciplinary pain rehabilitation program. SWEPPE has so far been tested as an extension of work-oriented interventions in healthcare, but it has not previously been tested in a more preventive context within an organisation.

There is currently a shortage of apps that facilitate self-management and promote collaboration and communication between employers and employees. SWEPPE addresses this gap by providing ongoing support to employees with musculoskeletal pain. Developed with the understanding that workplace interventions can benefit those working despite illness, SWEPPE aligns with the expanded responsibilities of employers, actively promoting sustained employment among individuals with pain. The goal is to create a healthier work environment, increase workforce participation and reduce societal costs by lowering sick leave rates. This evaluation aims to assess the experiences of employees dealing with pain, as well as their employers, in using SWEPPE on-site at the industrial workplace, and to explore how they specifically utilised the app.

## Methods

### Study design

This study has a mixed methods design where data was collected via a questionnaire, interviews and user data from the mobile application SWEPPE. This is a study that was carried out as part of a project at a large Swedish industrial company, Volvo Group Trucks Operations, Powertrain Production Köping (Volvo GTO PTP), aimed at enhancing understanding of how to improve working conditions and support a sustainable RTW/SAW for employees experiencing CMSP. Data was collected from October 2022 to May 2023 at the company's site in Köping, Sweden. As part of the project, participants had the possibility to use SWEPPE as a digital support tool for seven months, spanning from autumn 2022 to spring 2023. In this way, SWEPPE could also be tested in supporting individuals who remain in the labour market despite experiencing pain. The reporting follows the consolidated criteria for reporting qualitative research (CORE-Q).

### Participants

Participants were recruited using a convenience sampling strategy among employees participating in a project at Volvo GTO PTP. Inclusion criteria for employees to participate in the study comprised having contact with the human resources (HR) staff and the Occupational Health Care (OHC) at the company and participating in rehabilitation interventions at the workplace due to musculoskeletal pain persisting for more than three months. Participants could be currently working full time or be on sick leave. Additionally, employers responsible for the employees included in the study were also invited to participate in the study.

HR and OHC representatives convened an information meeting for employees who met the specified criteria. During the meeting, details about the study, the researchers and the SWEPPE digital support application were presented by the research team (MB, CT), and both employees and their employers were invited to participate in the study. Also, a lecture on pain from a biopsychosocial perspective and its impact on work was included. In this study, the term ‘employer’ encompasses individuals such as supervisors, managers and the physiotherapist (PT) from OHC.

### Intervention

**Figure 1.**
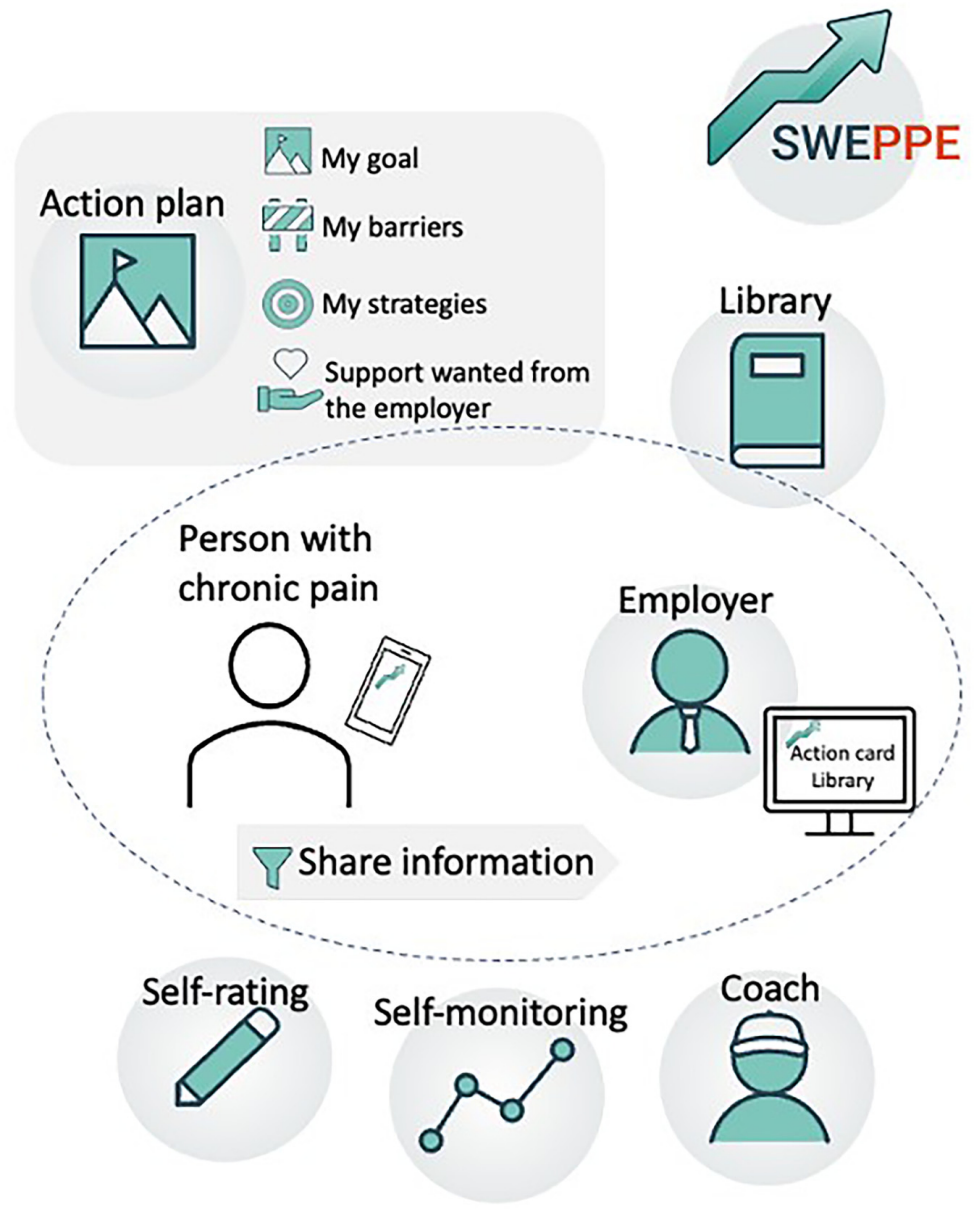
Functions in SWEPPE.

The intervention in this study involved the use of the SWEPPE smartphone application,^28^ which was provided as a supplement to the regular rehabilitation services offered by the OHC PT. Employees used SWEPPE on their smartphones, while employers, invited by their employees, accessed SWEPPE through a web application. SWEPPE has been described in detail elsewhere,^28^ however, key features of the SWEPPE app (Figure 1) include an action plan for RTW goals, outlining timelines, scope, and the identification of workplace obstacles, strategies, and support needs. Daily self-assessment and symptom monitoring enable personalised learning through the identification of patterns in data related to the unique set of variables the individual choose to register daily.^30^ Users can select variables to track, such as pain, sleep, activity balance, work situation, and stress, with the option to add custom variables. Assessment data can be reviewed over time to identify patterns. The app also provides direct communication with a coach where the user can ask a question related to management of chronic pain and RTW, and receive an answer in the app. The user also has access to a knowledge database (library) consisting of short texts and videos of topics referring to self-management of chronic pain and return to work. Users control what information is shared with employers through a sharing function; however, employers always have access to the library.

### Procedure

All participating employees were given a code to the SWEPPE smartphone application and instructions on how to open an account. The PT at the OHC was available to assist those who needed help to start up the use of SWEPPE. The employees decided if they wanted to invite their employer and what they wanted to share from the app.

The employees could also invite the PT and share information with her. While the PT is considered an employer representative, they are also actively involved in the rehabilitation process at the workplace. This allows the PT to facilitate communication and collaboration between the employer and the employees. The employees used SWEPPE to the extent they wanted during the study period of seven months. Additionally, they had individual regular follow-up meetings at the workplace with the PT, employers, and union representatives where data from SWEPPE were used for discussion and planning of the employee's work situation.

### Data collection

Data were collected through a questionnaire developed by the HR department based on their workplace survey, semi-structured interviews conducted by two of the researchers regarding employee and employer experiences, and user data obtained from the SWEPPE data base.

### Questionnaire

A questionnaire, administered by the HR representative, was sent out to all participants before the project started (Supplementary file 1). The questionnaire, developed specifically for the study, covered background questions, and open-ended questions regarding if the employees had any work-related and/or physical or psychological health conditions and if they would share information in SWEPPE with their employer.

### User data from the SWEPPE data base

User data of the 15 employees who had been using SWEPPE were collected from the SWEPPE user database. These data consisted of:

- Action plan: weekly updates of the employee's work-related goal, and registration of support required from the employer, updated monthly
- Self-monitoring: weekly evaluation of the employee's goal fulfilment, satisfaction and work ability
- Self-rating: if daily registration of at least one health aspect has been performed or not, indicated by yes/no
- Self-rating: registration of activity time distribution in different daily activities (sleep, paid work, household chores, exercise, other interests/activities, activities with children, taking care of relatives/older adults, voluntary work)
- Coach function: if and when the employee used the coach function in SWEPPE
- Time-use of SWEPPE: number of minutes spent using SWEPPE/day, and at what time of the day SWEPPE was used.

No user data were collected from the employers.

### Interviews

All participants were invited to the semi-structured interviews. The questions in the semi-structured interviews were carefully developed to address the key topics of interest but was not pilot-tested. They consisted of questions regarding participants’ experiences with SWEPPE's usability, functionality, and offered the submission of suggestions for improvement or potential feature removal. They were also asked about cooperation, focusing on how SWEPPE impacted communication and support. The interview questions explored how well SWEPPE facilitated workplace adaptations and enhanced understanding of employee's work-related changes. Finally, participants were asked to identify the stage in the RTW process where they felt SWEPPE was most needed (Supplementary file 2).

Semi-structured phone interviews were conducted by two of the researchers (CT, GML) with 13 employees, four employers, and one OHC PT in April 2023 and lasted approximately about 30 min. Field notes were written down during the interviews by the researcher conducting the interview. The participants were not known to the researchers prior to the study. The researchers in this project are occupational therapist with extensive experience and knowledge in conducting qualitative research including performing interviews and qualitative analysis, and clinical work in chronic pain management, RTW, digital support, and clinical interventions.

### Analysis

For ethical reasons and to protect the identification of participants in this small sample the open-ended questions from the questionnaire about health conditions and health issues were summarized on group-level by the HR-representative. The demographic data about the participants was then analysed using descriptive statistics.

User data from the SWEPPE user database were analysed using descriptive statistics. Data regarding employee support preferences from their employer, collected from the database, were grouped into categories based on similarities and differences.

**Figure 2.**
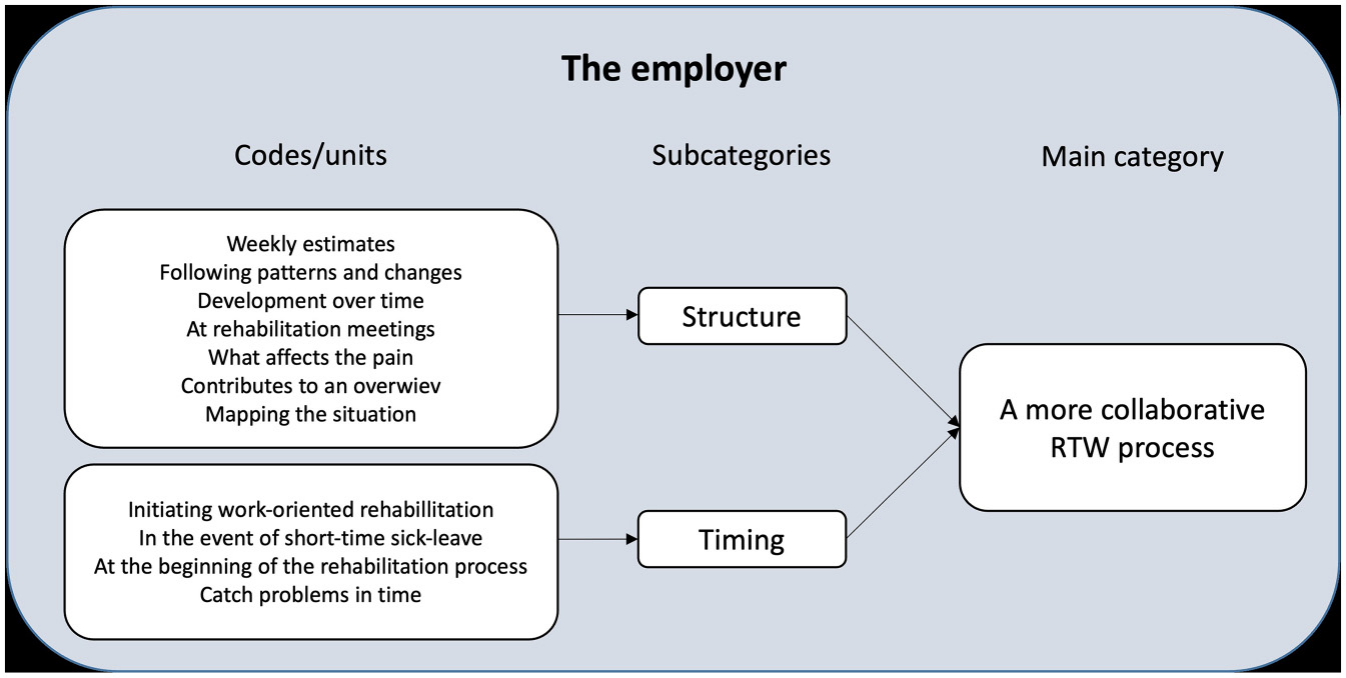
Extract from the analysis process. Examples related to the category A more collaborative process, from employer perspective.

Responses to the open-ended questions from the phone interviews were analysed by one of the authors (LK), in frequent dialogue with another researcher (GML), using a content analysis approach to identify core consistencies, as described by Patton.^32^ The other two authors (MB, CT) were also consulted on excerpts from the primary transcript data. Open coding of the material followed a structured process, where responses to each question were divided and categorised into units. These units consisted of words and sentences from the interviews.^33^ An example from the analysis process is written out in Figure 2. Quotations were included in the text to allow readers to evaluate the results, and they were transformed into written language in accordance with Kvale^34^ to prevent stigmatisation. In the qualitative part of the result section, brackets [] indicate implied words.

### Ethical approval

This study was conducted in accordance with the principles outlined in the Declaration of Helsinki.^35^ Participants were given both written and verbal information about the study, emphasising that their participation was voluntary and that they could withdraw at any point. All participants provided written informed consent. Data were treated confidentially and securely stored in a protected database. The study was approved by the Swedish Ethical Review Board (Dnr 2022-05812-02).

## Results

### Participants

Fifteen employees – eleven women and four men, aged 34 to 62 years – and ten employers, including a PT from OHC, provided informed consent to take part in the study. Each employer was responsible for overseeing one to three of the participating employees. Demographic information is available in Table 1.

**Table 1.** Demographic information of the study sample.

		Employees	Employers
Sex		n = 15	n = 10
	Men, n (%)	11 (73)	8 (80)
	Women	4 (27)	2 (20)
Education		n = 13	n = 5
	Elementary school (9 years)	2	0
	Upper secondary school (12 years)	11	3
	University	0	2
Age, m,range (SD)		53, 34–62 (±9,3)	41, 31–53 (±8,7)
Profession/Work assignment			
	Truckdriver	1	
	Logistician	1	
	Fitter/truckdriver	1	
	Fitter	2	
	Repairer	2	
	Machine operator	2	
	CNC operator	1	
	CNC operator/fitter	1	
	Operator/grinder	1	
	Operator	1	
	Head of department		2
	Supervisor		1
	Head of department/project leader		1
	Ergonomist/physiotherapist		1

The questionnaire showed that employees experienced various work-related physical issues affecting their hips, knees, feet, neck, shoulders, arms, elbows, and wrists. Reported musculoskeletal disorders included herniated discs in the neck and/or lower back, spinal conditions such as stenosis or vertebral displacement, osteoarthritis, and nerve entrapments like carpal tunnel syndrome. Additionally, employees reported high blood pressure and heart conditions. These physical issues led, according to the employees, to musculoskeletal pain, sleep disturbances, fatigue, challenges with walking or standing for extended periods, climbing stairs, and performing everyday tasks like placing dishes in a cupboard, cleaning, or scraping ice off a car windshield. The employees also claimed difficulties participating in physical activities and experienced heightened stress, worry, uncertainty and anxiety.

### User data from the SWEPPE application

The employees used a variety of functions in SWEPPE. The most used function was to perform daily registration of health aspects, performed by 14 of 15 participants (93%). Functions in the action plan, such as setting a work-related goal and performing weekly evaluations of the goal and work ability were used by 11 participants (73%), and registering support required from the supervisor was used by 12 (80%). Fourteen out of fifteen employees (93%) shared information with their supervisor. For details see Table 2.

**Table 2.** Overview of the employees’ (n = 15) use of the functions in SWEPPE during the project.

Function in SWEPPE	Description		
Action plan	Number of employees setting a work-related goal and performing weekly evaluation of the goal and work ability, n (%)		11 (73)
	Average number of weekly evaluations performed during the project, md (Iqr)		17 (3–26)
	Number of employees registering support needed from the supervisor, n (%)		12 (80)
	Average number of supports registered, md (Iqr)		2 (1–3)
		
Daily registration of health aspects	Number of employees performing daily registration of health aspects, n (%)		14 (93)
	Number of days with registration of at least one health aspect, md (Iqr)		173 (73–190)
	Number of employees performing daily registration of activity time distribution, n (%)		8 (53)
		
Share information	Number of employees sharing information with their supervisor, n (%)		14 (93)
	Number of employees sharing different types of information with the supervisors		
		RTW goal, n (%)	14 (93)
		Workplace obstacles, n (%)	12
		Workplace strategies, n (%)	12
		Support needs, n (%)	12*
		Graph over weekly evaluations, n (%)	12
Coach	Number of employees asking questions to the coach, n (%)		0 (=)
		

* One person did not use the app's sharing function with their employer but consistently shared information during planning meetings.

The employees’ registrations in SWEPPE of the support they required from their supervisors included assistance with work postures, breaks, workload, adapted work assignment, work pace, ergonomics and gaining understanding from colleagues (Table 3).

**Table 3.** Type of support the employees (n = 12) needed from the supervisors.

Category	Type of support	Number of employees, n
Work posture	Opportunity to regularly change working positions (standing/walking, sitting)	4
Work posture	To avoid situations that involve excessive bending, twisting the back or lifting from the floor	1
Work posture	Reducing/avoiding challenging work postures (uncomfortable postures or movements, forward bending, twisting, above shoulder height, below knee height)	3
Work posture	Reducing/avoiding uncomfortable grips, precision grips or repeated bending/twisting movements in arm or hand	2
Work posture	Avoiding repetitive tasks	2
Breaks	Opportunity to take short breaks and rest	5
Work load	Reducing/avoiding heavy lifting	4
Adapted work assignments	Avoiding the need to manage multiple tasks simultaneously	1
Adapted work assignments	Continuing with adapted tasks	1
Adapted work assignments	Limiting the amount of work performed in environments with extreme cold/draft/heat/noise/vibration	2
Work pace	Reducing work pace and avoiding stress	1
Ergonomics	Access to tools or work equipment individually adapted for the task	1
Knowledge and understanding from colleagues	Recognising that it's unacceptable to have colleagues who lacks the ability to behave appropriately and decent	1

On average, the employees spent 6.5 min, with a standard deviation of 9 min, in SWEPPE per day. SWEPPE was used around the clock with a peak during the evening. User statistics indicate that employees frequently engage in continuous learning, accessing an average of 28 articles or videos per week across various sections of the library, with a standard deviation of 14. Common topics viewed by the participants included ‘What is organisational and social work environment?’, ‘Strainful working postures’, ‘Work ability and realistic goals’, ‘Strain-related issues and women's work environment’, ‘What is activity balance?’, ‘Tips for an adapted activity pace’, ‘Tips and advice on sleep’, ‘Managing chronic pain’, ‘Web lectures on pain, and self-management strategies’ and ‘How to achieve acceptance? Listen to Jenny's story’.

### Interviews

**Figure 3.**
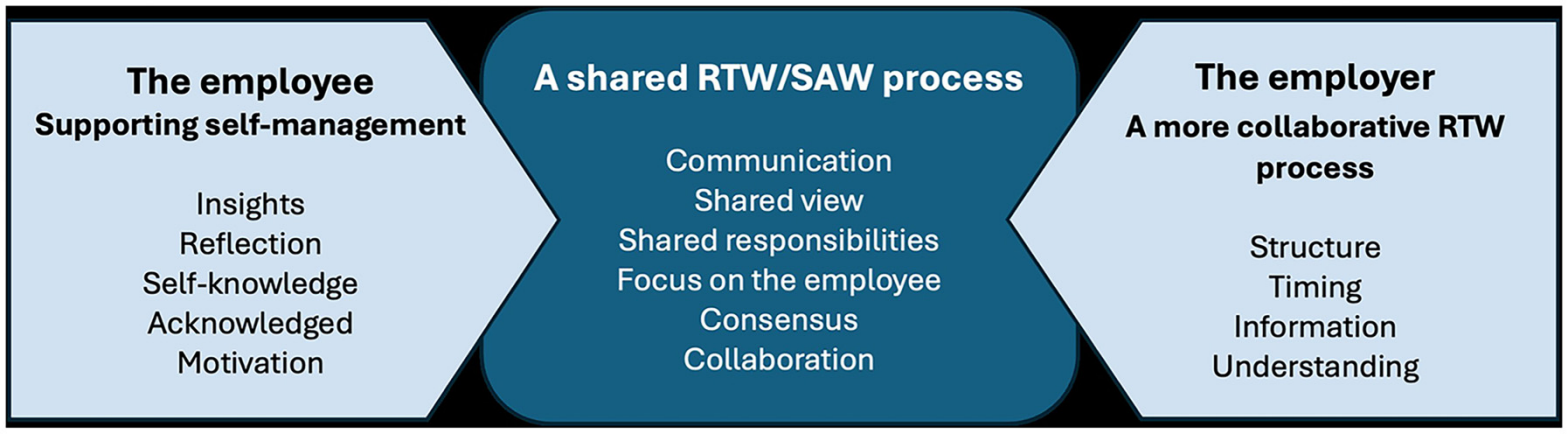
The combined experiences of employees and employers at VOLVO GTO PTP using SWEPPE contributed to supporting the return-to work/staying at work (RTW/SAW) process.

The results from the open-ended interview questions showed that, overall, the experiences of both employees and employers were interconnected within a shared RTW/SAW process. Summaries (the category A shared RTW/SAW-process) of the first two categories presented suggest that these shared experiences can positively impact the RTW process. The interaction between these three categories identified in the analysis is visually represented in Figure 3.

## The employee

### Supporting self-management

The monitoring function in SWEPPE gave valuable *insights* in how symptoms varied over time. Pain was important, but also sleep-pattern, daily activity-pattern and behaviours were registered. “… patterns between, for example, pain and sleep and also how I get worse in the weeks” (male, age 58). The employees valued using this monitoring to check daily status, identify periods with more intense symptoms, compare with previous periods and reflect on what in daily life and work situations might affect symptoms and behaviour. “… [monitoring] helped me to identify patterns in my pain-situation. I can see links between increased pain and… activities that I have performed” (male, age 56).

By monitoring symptoms, activities, and behaviours relevant to well-being and work performance, employees received continuous feedback over time, which encouraged *reflection.* “…I have been able to reflect over my days and activity balance” (male, age 56). Another employee stated: “It was good to write about how I felt and what I had done each day… I complained a bit, it became my wall of complaint. Really good actually” (female, age 61). In addition, SWEPPE offered graphical overviews which facilitated reflections as well as individual feedback and evaluation. When the variables were recorded in SWEPPE, an overview of the situation over time emerged which was seen as reliable - as facts, and this made it easier for the employees to rely on the associations that emerged in SWEPPE.

Monitoring gave the possibility to increase *self-knowledge* which was experienced as valuable in the RTW process. When employees gained a better understanding of their thoughts, emotions and reactions related to chronic pain and its effects, they found it easier to explain their situation to their employer and identify workplace situations that required adjustments.

“… I can look back and see – it is good to have knowledge [about their situation]. The manager helped me by asking how I feel… you collaborate more, it helps very much… now I will set a new goal to feel better and be able to work full time” (female, age 57).

Since information in the action plan, work ability and goal fulfilment could be shared with the employer during the process, the employer also had the chance to get a more nuanced view of the employees’ chronic pain and its effects on work. In some cases, this made the employer show a deeper understanding for the situation as a whole, which led to a sense of being trusted and *acknowledged* for the employee. “…a feeling that someone cares” (female, age 61). However, some employees felt hesitant to share the information in SWEPPE with their employer.

When the employer actively participated in the RTW-process and utilised the shared information in SWEPPE, employees felt a sense of engagement from their employer. This attention made employees feel acknowledged and valued.

“… the employer must be engaged. It can strengthen a person because you can easily be forgotten when you are on sick-leave. To feel this engagement, that you are important…” (female, age 46).

When this occurred, it boosted the employee's *motivation* to continue the process. Additionally, SWEPPE encouraged users to engage in an action-plan, which was particularly appreciated by employees, especially frequent users. “Most valuable were the goals, strategies and the daily ratings” (male, age 56). It enhanced their motivation to pursue new goals, and together with the self-rating and monitoring modules, it positively impacted their motivation. “… It is about holding and holding on [to the process]” (female, age 50).

## The employer

### A more collaborative RTW process

The use of SWEPPE in the RTW process allowed for a *structure* which was valuable and supported the process according to the participants. The weekly ratings made it possible to follow the employees patterns and changes, and this was important to identify critical situations for short-term actions and planning for long-term adaptations at the workplace. “ To get information over the week – then I can act in order to facilitate during the week so that my employee can cope…” (female, age 46). In the action-plan, the employee and employer could work together with goals, strategies and evaluation of adaptations - with the chronic pain in focus. “It has been an important tool and the person on sick-leave becomes central in the process. To see the development over time is really valuable” (female, age 53). The current situation and next steps became clearer, which was seen as a positive factor in progressing towards a sustainable work situation. However, it is possible to use SWEPPE in several ways, which is why it is important that the employer, as emphasised in the interviews, has a plan for how to use the app. In addition, employers might need instructions and support in how to apply SWEPPE as a structure for the RTW process.

The importance of using SWEPPE at *the right time* was highlighted by the employers. The app was most valuable in the beginning of the process, or when the employee went on sick-leave. “… at a new rehabilitation process, starting up with a new person. And even for people with a lot of short-term absence” (male, age 31). Another employer emphasised “ …to set up goals and strategies. And then a continuous follow-up” (female, age 46). The app assists with situation analysis, goal setting, strategy development, and follow-up, with earlier engagement being more beneficial. However, according to the employers in this study, once employees reach more than 30 working hours per week, the app seems to be less useful.

“I feel that at 75% [service] the interest in working with the estimates decreases, much is the same day by day. I see a difference in those I have been in regular contact with compared to others who have come further in their process. The app needs to be used together” (female, age 53).

Employers found that the comprehensive information provided by the SWEPPE app gave them a clearer view of their employees’ work situation. This *information* was highly valued because it not only offered valuable insights but also served as a foundation for discussions in rehabilitation meetings. “The information has been very valuable. I have had a clearer picture of the situation. We have used this information as grounds for discussion at our rehabilitation meetings” (male, age 35). Access to employees’ goals and strategies made the process smoother and discussions more productive.

Additionally, using SWEPPE helped employers gain a better *understanding* of how pain and related symptoms affected work ability, leading to greater awareness of the employee's challenges*. “*I have a clear understanding of how the pain is being impacted. Certain workstations have contributed to increased pain levels” (female, age 53).

This understanding made it easier to address issues and find sustainable solutions, resulting in a more participatory and collaborative process.

### A shared RTW/SAW-process

The experiences of employees and employers in using SWEPPE influenced the overall RTW-process. *Communication* between the employee and employer was often facilitated due to the structure and the shared information in the app which were updated regularly. This led to a *shared view* of the situation and further, according to the participants, a basis for *shared responsibility* where both the employee and employer were important for progress towards a sustainable work situation.

“SWEPPE has been used during regular meetings with the rehabilitation group we've had. SWEPPE has played a central role in these meetings, allowing us to reflect on how I feel and how things are going for me. Now, we need to find an alternative that works better within her work situation. The union representative played an important role and became concerned based on what he observed in SWEPPE” (employee, female, age 54).

In terms of sick-leave, the primary *focus was on the employee*, who became the central person in the process. The employees reported being treated seriously, and actively involved in evaluating and discussing progress, adaptations and solutions. The use of SWEPPE often resulted in increased engagement and an increased sense of importance for the employee.

“It [SWEPPE] is a crucial tool that places the person more at the center. Observing the development over time is incredibly valuable” (employer, female, age 53).

Reaching a *consensus* about the situation and the process forward is beneficial and a good prerequisite for *collaboration* between the employee and employer. When a collaborative state was achieved it led to positive experiences for both the employee and employer, a feeling of working together resulted in deeper engagement and perseverance in the RTW/SAW-process. Thus, employees and employers reported that SWEPPE supported the RTW-process in a positive way.

“We have used this as a foundation for discussion, with the information presented clearly in black and white, showing what things look like. It allows me to track the workload and see how the pain increases or decreases” (employer, male, age 35).

However, it was emphasised that these positive aspects relied on an engagement from the employer and sense of possibilities from the employee. Some employees experienced time constraints, poor communication, and limited opportunities to influence decisions or propose solutions. In these cases, SWEPPE did not contribute to progress in the RTW-process.

“Everyone is overwhelmed and there's no time, yet the employer must be involved. It can empower you, as a sick leave employee, to feel valued and committed. But it's sad that there's no time for meaningful connection anymore” (employee, female, age 46).

In terms of suggestions for improving the app, employers expressed a desire for real-time access to the SWEPPE app, allowing them to monitor changes in employees’ pain levels and other symptoms as they happen via notifications in, for example, their computers/mobile phones. This immediate visibility would enable employers to proactively implement supportive measures at the earliest signs of discomfort, aiming to prevent escalation that could lead to sick leave. By addressing symptoms early, employers hope to support employees in maintaining consistent work attendance and well-being, ultimately reducing the likelihood of extended absences and fostering a more responsive, health-conscious work environment.

“I’d like to see more of this information, as it's valuable to me in my role as a supervisor. Does the situation tend to worsen after a weekend? Does it increase in specific work situations? It would be helpful to have daily estimates so I can stay involved. A phone call or email in the evening would allow me to have strategies ready for the next day” (employer, male, age 42).

## Discussion

This study aimed to assess the experiences of employees dealing with musculoskeletal pain, as well as their employers, in using SWEPPE on-site at the industrial workplace, and to explore how they specifically utilised the app. Focusing on individuals who continue to work despite their pain (presentees) holds significant value for both the individual and society, as they are more likely to respond positively to workplace interventions, stay employed and return to full duties.^10^ Workplace interventions have been shown to positively impact outcomes such as employment status and work ability,^36–38^ underscoring their importance. While support from employers or supervisors in work adaptation is essential, employees experiencing pain often lack adequate support,^15^ frequently because employers may not have the necessary knowledge to fulfil these responsibilities effectively.^16^

Not all employees in this study were comfortable sharing health information with their employers, even though such transparency could improve the employer's understanding of the employee's needs, potentially leading to more tailored and effective workplace interventions. Also, previous studies on SWEPPE^30,31^ have shown that reluctance to share information may stem from a perceived lack of interest or commitment from the employer. To address this challenge, it may be helpful to highlight alternative avenues for sharing sensitive information. For instance, if employees feel a lack of trust towards their direct supervisor, they could consider sharing updates with a safety representative, HR personnel or someone in the OHC department instead. These options offer employees more control over their information-sharing decisions, fostering a sense of safety while still enabling valuable insights that could contribute to a more supportive and responsive work environment.

The findings highlight several key aspects of the intervention's impact on the RTW/SAW processes. The SWEPPE app positions the employee at the centre of the RTW process, providing tools for them to actively monitor and reflect on their health symptoms. This monitoring feature not only offers employees greater insight into their condition but also encourages self-reflection, which fosters self-management. A key factor in the success of digital health support tools is their ability to empower individuals by enabling them to take control of their health. By focusing on empowerment and self-management, tools like SWEPPE can enhance the individual's motivation and capability to independently manage their symptoms effectively.^22^

A notable strength of the SWEPPE app lies in its self-assessment monitoring feature, which presents a comprehensive overview of symptoms, allowing employees to track patterns over the past week or month. This continuous self-assessment empowers employees to identify trends and adjustments that may be necessary for managing their work and health needs more sustainably.^28^

Further, one of the standout findings is that implementation of the SWEPPE app in the workplace significantly enhanced the RTW/SAW process by promoting transparent, ongoing communication between employees and employers. The app created a structured platform where both parties could regularly exchange updates, resulting in a shared understanding of the employee's situation and needs. This shared understanding cultivated a sense of employer responsibility, essential for fostering a sustainable work environment. This shared insight broadens perspectives and informs adjustments that can enhance the work environment sustainably. Additionally, support and collaboration are highlighted as critical elements in maintaining long-term workplace inclusion, particularly for employees with ongoing health challenges.^19,21^

SWEPPE's collaborative foundation supports the necessary cooperation among all parties to develop effective workplace strategies and adaptations. In this study, the app's most frequently used functions among employees included setting work-related goals and performing weekly evaluations of these goals and their work ability, with 73% of employees engaging in these activities. According to Kruse,^22^ the opportunity to formulate an individual goal e.g., in terms of time and extent for return to work is significant to the success of digital support. Additionally, almost all employees in this study regularly tracked their health through daily registrations, underlining a strong commitment to self-monitoring. This confirms that self-monitoring of symptoms and self-tailoring of strategies are valued functions in apps for self-management of chronic pain.^39^

The information most shared with employers included RTW goals, workplace obstacles, proposed strategies and specific support needs. Employees also frequently shared visual summaries, such as graphs of their weekly evaluations, which provided a clear overview of their progress and challenges. This shared information facilitated mutual understanding and allowed for timely, informed interventions to support the RTW/SAW process.

The study sample consisted of people ranging in age and with various work-related physical issues including neck and shoulder complaints. Many of these complaints are associated with material handling, vibration, or demanding work positions.^40^ Job demands often lack the flexibility needed to optimise productivity, health, and safety, regardless of age or chronic disease.^41^ According to Main et al.,^42^ an important form of support for workers experiencing pain is the provision of temporary or permanent changes in the work situation, for example work rotation, reorganisation of workstations and, modified hours or duties. This is confirmed by the examples of support required from the employer, as identified by the employees, regarding adaptation of the work situation. Employees requested several types of support to help manage their work more effectively. Key areas included guidance on proper work posture, regular breaks and adjustments to their workload. They also sought adapted work assignments and a manageable work pace to help them perform tasks comfortably and sustainably. Social support is an equally important yet often underappreciated component in moderating job-related stress, whether it is provided on an individual level, within a working group, or at an organisational level.^42^ In our study, employees expressed a need for understanding and empathy from colleagues, which they felt would foster a more supportive and inclusive workplace environment. This underscores the significant role that social groups within the workplace play in accommodating or mitigating the impact of disabling health conditions.^42^ Overall, these types of support collectively contribute to a work setting that respects individual health needs and promotes long-term participation.

Previous results from testing the SWEPPE app with patients who completed a rehabilitation programme^30,31^ indicated that the app was user-friendly, with an intuitive interface that made it easy to navigate. Users found the app relevant, valuable, and supportive in enhancing their knowledge and strategies for managing their condition. Additionally, it facilitated constructive collaboration between employees and employers.^30,31^ These positive findings are reaffirmed in the current study, further validating SWEPPE's usefulness as a supportive tool in the RTW/SAW process, even in an industrial setting. In terms of suggestions for improving the app, we are currently investigating the technical possibilities for a more proactive support function, enabling real-time access and monitoring of changes in employees’ reporting of distress. Based on the findings of our study, we plan to investigate this further.

The timing of a self-management app is crucial, and individuals need to feel validated before they will engage with a self-management app. Introducing such an app early in a treatment process can be particularly beneficial.^43^ This is supported by the findings where both employees and employers highlighted the importance of using the SWEPPE app at strategically chosen times to maximise its impact. They agreed that the app proved most beneficial at the start of a rehabilitation case or in instances of frequent short-term sick leave, where early intervention and monitoring are crucial. Additionally, they found that the app was especially effective for employees working fewer than 30 h per week, as these part-time schedules may require more tailored support to ensure consistent attendance and to facilitate gradual, sustainable reintegration into the workplace.

## Strengths and limitations

A strength of this study is the combination of open-ended questions and user data from the app, which provides a comprehensive view of participants’ opinions and their app usage.

In qualitative research, ensuring credibility, dependability, and transferability is essential for establishing trustworthiness.^44,45^ Dependability in particular, is enhanced where the research process and data analysis methods are thoroughly detailed.^45^ The study's limitations include a relatively small sample size and its focus solely on a Swedish context. This may restrict the transferability of the findings, as the results may not fully represent the experiences and needs of a broader, more diverse workforce. Consequently, further research with a larger and more gender-balanced sample would be beneficial to validate and expand on these findings. Despite the small sample size in this study, the participants’ diverse experiences may enhance the credibility of the findings.^44^ Strengths of the study is that interviews were performed by researchers not connected to the employers at the industrial company which can increase the participants willingness to share both positive and negative experience. Further, user data collected in SWEPPE was also separate from the company. Throughout all stages of the study, the researchers remained conscious of their preexisting knowledge and experiences in the field. Data analysis was conducted in collaborative work process to ensure that findings were grounded in data.

Background data about the study participants was collected with a questionnaire developed and administrated by the HR-department specific for the study. Thus, the questionnaire was not a validated tool for assessment of health status or work ability. Using a standardized questionnaire regarding could have provided more detailed background information about the participants.

Another potential limitation is the possibility of selection bias, as the participants may have been individuals who are already more positive and comfortable with technology. This could mean that the study results reflect a more favourable view of the app than might be observed in a more varied group, including those who are less familiar or less enthusiastic about digital tools. Future studies could aim to include participants with a broader range of attitudes and technical skills to provide a more comprehensive understanding of the app's usability and effectiveness across different user profiles.

To further improve the potential of SWEPPE, future research should focus on enhancing employer engagement and ensuring they are adequately trained to use these tools effectively. Addressing barriers faced by employees, such as time constraints and communication challenges, is also crucial. Longitudinal studies could provide deeper insights into the long-term impact on RTW/SAW outcomes. Additionally, expanding the use of SWEPPE to different workplace settings and populations could help generalise the findings and identify best practices for broader implementation.

## Conclusions

Overall, the study indicates that SWEPPE can be a valuable tool in supporting the RTW/SAW process for employees with musculoskeletal pain, even in an industrial setting. Its success relies on active participation from both employees and employers, fostering a collaborative and supportive work environment. Effective communication, early and active engagement from employers and the empowerment of employees through self-management are crucial for its success. Addressing identified challenges and ensuring proper implementation and support can further enhance its effectiveness, making it a valuable asset in creating sustainable work environments.

## Supplemental Material

sj-docx-1-dhj-10.1177_20552076251342014 - Supplemental material for Evaluation of the clinical use of a digital support app for employees with musculoskeletal pain and their employers in an industrial workplace. A mixed methods study Supplemental material, sj-docx-1-dhj-10.1177_20552076251342014 for Evaluation of the clinical use of a digital support app for employees with musculoskeletal pain and their employers in an industrial workplace. A mixed methods study by Gunilla M Liedberg, Mathilda Björk, Linn Karlsson and Christina Turesson in DIGITAL HEALTH

sj-docx-2-dhj-10.1177_20552076251342014 - Supplemental material for Evaluation of the clinical use of a digital support app for employees with musculoskeletal pain and their employers in an industrial workplace. A mixed methods study Supplemental material, sj-docx-2-dhj-10.1177_20552076251342014 for Evaluation of the clinical use of a digital support app for employees with musculoskeletal pain and their employers in an industrial workplace. A mixed methods study by Gunilla M Liedberg, Mathilda Björk, Linn Karlsson and Christina Turesson in DIGITAL HEALTH

## Abbreviations

HR: Human Resources
OHC: Occupational Health Care
PT: physiotherapist
RTW: return-to-work
SALAR: Swedish Association of Local Authorities and Regions
SAW: staying-at-work
SWEPPE: Sustainable WorkEr, a digital support for Persons with chronic Pain and their Employers
Volvo GTO PTP: Volvo Group Trucks Operations, Powertrain Production
