# Supplementary material for: Evaluation of the clinical use of a digital support app for employees with musculoskeletal pain and their employers in an industrial workplace. A mixed methods study
Source: Digit Health. 2025 May 14;11:20552076251342014. doi: 10.1177/20552076251342014 (PMC12078973; doi:10.1177/20552076251342014)
Supplement: sj-docx-1-dhj-10.1177_20552076251342014 - Supplemental material for Evaluation of the clinical use of a digital support app for employees with musculoskeletal pain and their employers in an industrial workplace. A mixed methods study [file sj-docx-1-dhj-10.1177_20552076251342014.docx]

S1. Content of the questionnaire

| Participant | Question | Response option |
| --- | --- | --- |
| Employer | - Age | Years |
|  | - Sex | Man, woman |
|  | - Education | Elementary school, upper secondary school, university |
|  | - Profession/work assignment | Open-end question |
| Employee | - Age | Years |
|  | - Sex | Man, woman |
|  | - Education | Elementary school, upper secondary school, university |
|  | - Profession/work assignment | Open-end question |
|  | - Do you have any work-related physical issues? Please describe | Open-end question |
|  | - How do these physical issues affect you? Please describe | Open-end question |
|  | - Do you have any physical health conditions? Please describe | Open-end question |
|  | - Are you willing to share information with your supervisor? | Yes, no |
