# Supplementary material for: Evaluation of the clinical use of a digital support app for employees with musculoskeletal pain and their employers in an industrial workplace. A mixed methods study
Source: Digit Health. 2025 May 14;11:20552076251342014. doi: 10.1177/20552076251342014 (PMC12078973; doi:10.1177/20552076251342014)
Supplement: sj-docx-2-dhj-10.1177_20552076251342014 - Supplemental material for Evaluation of the clinical use of a digital support app for employees with musculoskeletal pain and their employers in an industrial workplace. A mixed methods study [file sj-docx-2-dhj-10.1177_20552076251342014.docx]

S2. Content of the semi-structured interviews and examples of questions and follow-up questions.

| Topic | Questions |
| --- | --- |
| Functions in SWEPPE | Was SWEPPE useful for you as an employer/employee? In what way? Can you give an example |
|  | Which parts of SWEPPE have been most valuable to you? Why, why not? How? |
|  | Do you think any parts could be added or that some existing parts could be developed (or removed) to make SWEPPE a better support tool? Why, why not? How? |
| Cooperation | How have you used SWEPPE in communication with your supervisor/employee? Why, why not? |
|  | How has SWEPPE affected or changed communication with your supervisor/employee? |
|  | Have you received/provided a better support in your/the employee’s work situation by using SWEPPE? Why, why not? How? |
|  | Has your contact with your supervisor/employee increased by using SWEPPE? Why, why not? How? |
|  | Has the collaboration between you and your supervisor/employee regarding workplace adjustments (or other interventions) been facilitated by using SWEPPE? Why, why not? How? |
|  | Do you feel that SWEPPE has helped you gain greater understanding from your supervisor/for your employee regarding difficulties or for managing the work situation? Why, why not? How? |
|  | When do you perceive the need for SWEPPE to be the greatest? Why? |
